# A Study to Investigate the Safety and Immunogenicity of Monovalent Omicron LP.8.1-Adapted BNT162b2 COVID-19 Vaccine in Adults ≥ 65 Years of Age and High-Risk Adults 18–64 Years of Age (Preliminary Results)

**DOI:** 10.3390/vaccines14040350

**Published:** 2026-04-15

**Authors:** Rucha Dadhe, Juleen Gayed, Muneeb Iqbal, Rohit Solan, Han Wu, Hua Ma, Xia Xu, Federico J. Mensa, Todd Belanger, David Cooper, Robin Mogg, Annaliesa S. Anderson, Özlem Türeci, Uǧur Şahin, Pirada Suphaphiphat Allen, Kayvon Modjarrad, Alejandra Gurtman, Kelly Lindert

**Affiliations:** 1 Pfizer Vaccines, Pfizer Ltd., Marlow SL7 1YL, UK; rucha.dadhe@pfizer.com (R.D.);; 2 Pfizer Vaccines, Pfizer Inc., Collegeville, PA 19426, USA; 3 BioNTech US Inc., Cambridge, MA 02139, USA; 4 Pfizer Vaccines, Pfizer Inc., Pearl River, NY 10965, USA; 5 BioNTech, 55131 Mainz, Germany; 6 Pfizer Vaccines, Pfizer Inc., Cambridge, MA 02139, USA

**Keywords:** BNT162b2, COVID-19, Omicron, SARS-CoV-2, vaccine, variant-adapted

## Abstract

Background/Objectives: This study evaluated the Omicron LP.8.1 variant-adapted BNT162b2 mRNA vaccine (LP.8.1-adapted BNT162b2). Methods: This analysis is part of an ongoing phase 3 open-label study evaluating the immunogenicity, safety, and tolerability of LP.8.1-adapted BNT162b2. Reported here are descriptive 2-week post-vaccination results in 18–64 -year-olds at high risk of severe COVID-19 and in ≥65-year-olds who received the Omicron KP.2-adapted COVID-19 vaccine ≥ 6 months previously. Primary immunogenicity endpoints included neutralizing antibody geometric mean titers (GMTs) against LP.8.1 and KP.2 at 2 weeks after vaccination and geometric mean fold rises from baseline to 2 weeks after vaccination. Results were compared with a historical control group of adults who received KP.2-adapted BNT162b2 in a previous study. Tolerability and safety were also assessed. Results: Overall, 104 participants received LP.8.1-adapted BNT162b2 (18–64-year-olds, *n* = 51; ≥65-year-olds, *n* = 53). Baseline neutralizing GMTs were higher in LP.8.1-adapted BNT162b2 recipients than in the historical control group of KP.2-adapted BNT162b2 recipients against both sublineages (248 vs. 157 against LP.8.1; 372 vs. 187 against KP.2). Serum-neutralizing LP.8.1 and KP.2 GMTs increased 2 weeks after vaccination with LP.8.1-adapted BNT162b2 (1752 against LP.8.1; 2104 against KP.2) and historical control groups (1555 and 2395, respectively), and across both age groups. Reactogenicity events with LP.8.1-adapted BNT162b2 were generally mild or moderate and occurred at generally similar frequencies in both age groups. Adverse events were reported in 4.8% of participants (all in 18–64-year-olds); no serious adverse events were reported. Conclusions: After 2 weeks of follow-up, and in a small sample size, LP.8.1-adapted BNT162b2 was immunogenic in ≥65-year-olds and ≥18-year-olds at high risk of severe COVID-19. The safety and tolerability profile for LP.8.1-adapted BNT162b2 was consistent with the current US prescribing information for BNT162b2 and that of other variant-adapted BNT162b2 vaccines (Clinicaltrials.gov Identifier: NCT07069309, registered 16 July 2025).

## 1. Introduction

COVID-19, caused by infection with SARS-CoV-2, remains a public health concern, particularly among vulnerable populations, such as older adults and individuals with certain comorbid medical conditions [1,2,3]. The risk of COVID-19-associated hospitalization and/or death is substantially increased in individuals with comorbidities including pulmonary, cardiovascular, and kidney disease, obesity, diabetes, dementia, and stroke [4,5,6].

Since the onset of the COVID-19 pandemic in 2020, SARS-CoV-2 has continued to evolve, with antigenically divergent variants and evolution from pandemic to endemic waves of disease [7,8]. Beginning in December 2021, the Omicron variant and its sublineages became predominant globally and were shown to exhibit a high level of immune escape from both vaccine-induced and infection-induced immunity [9,10,11].

With the continued evolution of SARS-CoV-2, vaccination is key to minimizing the risk of severe disease, including death resulting from COVID-19 [12]. Cohort studies support the benefits of COVID-19 vaccination in reducing the risk of severe outcomes. For instance, in a cohort study of 28.6 million French adults 18 through 59 years of age, 21% of whom were unvaccinated, individuals who had received a COVID-19 mRNA vaccine had a 74% decreased severe COVID-19-associated and no increased all-cause mortality risk compared with age-matched unvaccinated individuals over a 4-year period [13]. Additionally, in a whole-of-population analysis of 3.9 million Australians ≥ 65 years of age, 5% of whom were not vaccinated against COVID-19, vaccine effectiveness (VE) against COVID-19-specific and all-cause mortality during a period that included SARS-CoV-2 Omicron infections ranged from 73% to 93% [14]. VE varied by number of doses, time period during the Omicron wave, and time since receipt of most recent vaccine dose, with considerable waning of VE to 34% at 6 months after vaccination. These cohort analyses are supported by a retrospective surveillance study of the World Health Organization European region, which estimated that vaccination reduced the number of COVID-19-related deaths over a 2.5-year period between December 2020 and March 2023 by 59% (1.56 million lives saved out of an expected 2.62 million deaths), with 60% of this mortality reduction occurring after the emergence of the Omicron variant [12]. Additionally, a comparative effectiveness study estimated that by October 2024, vaccination against COVID-19 prevented approximately 2.5 million deaths worldwide, with vaccination saving an estimated 1 life-year per 900 doses [15,16]. It is anticipated that vaccination will continue to reduce severe outcomes associated with new lineages, particularly in older adults and other high-risk individuals [8].

BNT162b2 is an mRNA-based vaccine, which is approved for the prevention of COVID-19 caused by SARS-CoV-2 [17], the original vaccine encoded for the ancestral SARS-CoV-2 strain spike glycoprotein (i.e., Wuhan-Hu-1) [18]. Booster doses with the original BNT162b2 initially provided some measure of protection, but this was not sustained against the antigenically shifted Omicron strain [19,20]. Consequently, updated variant-adapted bivalent COVID-19 mRNA vaccines targeting emerging Omicron subvariants (BA.1; BA.4/BA.5) alongside the ancestral strain were introduced in 2022 [21,22]. Monovalent Omicron JN.1 lineage-adapted vaccines were subsequently introduced in the United States and Europe [23,24]. Since 2022, different variant-adapted versions of the BNT162b2 vaccine have been approved, encoding Omicron XBB.1.5, JN.1, and KP.2 [25,26]. In August 2024, the US Food and Drug Administration (FDA) recommended the KP.2 sublineage as the preferred variant composition of the COVID-19 vaccine formula for the 2024–2025 season [27]. In early 2025, the Omicron LP.8.1 lineage began to spread globally; LP.8.1 is antigenically distant to KP.2 with nine mutations in the spike protein compared with JN.1, and represents an antigenic drift rather than a shift, as was observed from XBB to JN.1 [28,29,30,31].

As SARS-CoV-2 continues to evolve antigenically distinct lineages [7], real-world evidence has consistently demonstrated that more closely antigenically matched vaccines improve protection against COVID-19 [32]. The FDA and European Medicines Agency advised that COVID-19 vaccines for use beginning in the autumn of 2025 should be monovalent JN.1-lineage-based vaccines, preferentially using the LP.8.1 sublineage [23,33]. Additionally, given that older adults and those with certain comorbidities are at high risk of severe COVID-19 [34,35,36,37], vaccination is particularly important in these individuals; it is thus important to investigate the immunogenicity, safety, and tolerability of variant-adapted vaccines in these populations. Therefore, we evaluated the monovalent Omicron LP.8.1 variant-adapted BNT162b2 mRNA vaccine among older adults and adults 18–64 years of age at high risk of severe illness. Here, we report 2-week post-vaccination immunogenicity and safety and tolerability results.

## 2. Materials and Methods

### 2.1. Study Design

This open-label analysis, which is part of an ongoing phase 3 study (ClinicalTrials.gov Identifier: NCT07069309; registered 16 July 2025), is evaluating the immunogenicity, safety, and tolerability of the Omicron LP.8.1 variant-adapted BNT162b2 mRNA vaccine in individuals ≥ 12 years of age. The study includes 2 cohorts, each based on age group and high risk of severe COVID-19. Reported here are the results for participants in the first cohort, which enrolled participants into 2 age groups: individuals 18–64 years of age who have at least 1 risk factor for severe COVID-19 and those ≥65 years of age. Participants in this cohort were enrolled at 5 sites in the United States from 8 to 9 July 2025, with a data readout of 24 July 2025 for the 2-week data reported here.

Eligibility criteria for this cohort were age 18 years and older; receipt of an Omicron KP.2-adapted COVID-19 vaccine more than 6 months before study enrollment; and no other COVID-19 vaccine or physician-confirmed COVID-19 since that vaccination up to enrollment. Participants 18–64 years of age were required to have a least 1 risk factor for severe COVID-19, as shown in Table S1, from risk factors listed by the Centers for Disease Control and Prevention [38], which were to be clinically stable for at least 6 weeks before enrollment. Participants were excluded if they had a history of severe adverse reaction associated with vaccination; bleeding diathesis or a condition associated with prolonged bleeding; were immunocompromised; pregnant or breastfeeding; or had a history of cardiomyopathy, myocarditis, or pericarditis. Additional exclusion criteria are described in the Supplementary Materials.

The ethical conduct of this clinical trial is described in the Supplementary Materials. All participants provided written informed consent before undergoing any study-specific procedures.

### 2.2. Intervention, Endpoints, and Assessments

Participants received a single injection of the LP.8.1-adapted BNT162b2 vaccine at the 30 µg dose level into the deltoid muscle (preferably of the nondominant arm) at a study site clinic by a trained and qualified member of the study staff.

Description of humoral immune responses to LP.8.1-adapted BNT162b2 at 2 weeks after vaccination was the primary immunogenicity objective. Serum samples for immunogenicity assessments were collected before vaccination (i.e., at baseline; Day 1) and again at 2 weeks after vaccination with LP.8.1-adapted BNT162b2 or placebo. Additional serum samples will be collected at 1 month and 6 months after vaccination. Samples were tested against Omicron LP.8.1 and Omicron KP.2 viruses using a validated recombinant SARS-CoV-2 neutralization assay (described previously [39,40]). Briefly, serially diluted serum was incubated with virus in a 384-well plate and then transferred to a cell monolayer overnight. Infection by non-neutralized virus was detected by counting green fluorecent viral foci with a cell-imaging reader. The titer is the reciprocal dilution at which 50% of the virus is neutralized. Immunogenicity endpoints described in this report include neutralizing antibody geometric mean titers (GMTs) 2 weeks after vaccination, geometric mean fold rises (GMFRs) from baseline to 2 weeks after vaccination, and percentages of participants with seroresponse 2 weeks after vaccination (seroresponse is defined in the Supplementary Materials). Immunogenicity endpoints include comparison with an historical control group of adults from a previous phase 2/3 study (NCT05997290) who received Omicron KP.2-adapted BNT162b2 at least 150 days after any previous COVID-19 vaccination [41]. Participants from the historical control group were from the immunogenicity evaluable population in the previous study and included participants ≥ 65 years of age, as well as those 18–64 years of age who had one or more medical conditions associated with an increased risk of severe COVID-19; participants were identified by retrospective review of medical history reported at enrollment.

The safety objective was to describe LP.8.1-adapted BNT162b2 safety and tolerability. The percentage of participants reporting local reactions (i.e., pain at the injection site, swelling, and redness) and systemic events (i.e., fever, fatigue, headache, chills, vomiting, diarrhea, and new or worsened muscle or joint pain) through 7 days following vaccination by severity grading was determined (severity grading is shown in Table S2 [42]). Participants were required to complete a daily reactogenicity electronic diary (e-diary) to record local and systemic events over the 7-day collection period (or longer for ongoing symptoms). Adverse events (AEs) and serious AEs (SAEs), which were categorized by Medical Dictionary for Regulatory Activities (version 28.0) preferred terms and system organ classes, were to be recorded from vaccination until 1 month after vaccination and until 6 months after vaccination, respectively. Protocol-specified AEs of special interest (AESIs) included confirmed diagnosis of myocarditis or pericarditis, as well as potential menstrual cycle disturbances occurring within 6 months after vaccination. Reported here are safety endpoints through 2 weeks after vaccination. Participants 18–64 years of age at high risk of developing severe COVID-19 also underwent additional pre-vaccination assessments, including electrocardiogram, blood pressure, and pulse rate measurements as outlined in the Supplementary Materials.

### 2.3. Statistics

Statistical analyses were performed using SAS^®^ (version 9.4; SAS Institute, Cary, NC, USA). All statistical analyses are descriptive, with no formal hypothesis testing.

Immunogenicity analyses were conducted on the evaluable immunogenicity population (Table S3). There was no imputation of missing serology results. Immunogenicity results below the lower limit of quantitation (LLOQ) were set to 0.5 × LLOQ in the analysis; an exception was if the baseline assay result was less than the LLOQ, and the post-vaccination result was at or greater than LLOQ, in which case the baseline value was set to LLOQ for the fold rise calculation. GMTs and GMFRs were calculated as the means of the logarithmically transformed neutralizing titers and means of the difference in the logarithmically transformed neutralizing titers (later time point minus earlier time point, respectively) and exponentiating the mean to express the results on the original scale. Associated 2-sided 95% CIs for GMTs and GMFRs were obtained by constructing the CIs using Student’s *t* distribution for the mean and mean of the difference in the neutralizing titers in the logarithmic scale and exponentiating the confidence limits. Seroresponse rates are presented as the percentages of participants with seroresponse with associated Clopper–Pearson 95% CIs.

All participants who received LP.8.1-adapted BNT162b2 were included in the safety analyses (i.e., the safety population; Table S3). Descriptive statistics are provided for categorical safety and tolerability variables, including the percentages, numerators, and denominators.

## 3. Results

### 3.1. Study Conduct and Participants

Overall, 104 participants were enrolled in this cohort (18–64 years old, *n* = 51; ≥65 years old, *n* = 53), and all participants received the LP.8.1-adapted BNT162b2 vaccine (Figure S1). At the time of analysis, 50 and 53 participants in the 18–64 and ≥65 years of age groups, respectively, had completed the 2-week post-study vaccination visit. No participants had withdrawn from this study at the time of the analysis.

Participant demographics and baseline characteristics for the safety population are shown in Table 1. In the 18–64 and ≥65 years of age groups, 33.3% and 56.6% of participants, respectively, were male, 78.4% and 84.9% were White, and the median age at vaccination was 54.0 and 73.0 years. Overall, 94.1% and 84.9% of participants in the 18–64 and ≥65 years of age groups were positive for SARS-CoV-2 at baseline. The median time since the last dose of COVID-19 vaccine (received before this study) to the study vaccination was 10.0 months in the 18–64 years of age group and 9.6 months in the ≥65 years of age group. The percentages of individuals who were considered obese (body mass index ≥ 30.0 kg/m^2^) at enrollment were 58.8% in the 18–64 years of age group and 32.1% in the ≥65 years of age group. Metabolism and nutrition disorders formed the most commonly reported system organ class for medical history (74.5% and 88.7% of participants in the 18–64 and ≥65 years of age groups, respectively; Table S4). The most frequently self-reported medical history preferred terms were obesity (47.1%) and COVID-19 (43.1%) in the 18–64 years of age group and hypertension (35.8%) and COVID-19 (30.2%) in the ≥65 years of age group.

Participant demographics and baseline characteristics of the evaluable immunogenicity study population and the historical control population (KP.2-adapted BNT162b2) are shown in Table S5. The median time since previous COVID-19 vaccination was shorter in the current study (9.5 months and 9.3 months in participants 18–64 and ≥65 years of age, respectively) compared with the historical control group (13.3 months and 21.4 months, respectively).

### 3.2. Immunogenicity

Overall, 83 participants who received the LP.8.1-adapted BNT162b2 vaccine (18–64 years of age, *n* = 37; ≥65 years of age, *n* = 46) and 74 individuals from the historical control group (KP.2-adapted BNT162b2; 18–64 years of age, *n* = 41; ≥65 years of age, *n* = 33) were included in the evaluable immunogenicity population. Among participants in the LP.8.1-adapted BNT162b2 vaccine group, 21 were excluded from the evaluable immunogenicity population because of important protocol deviations (18–64 years of age, *n* = 14; ≥65 years of age, *n* = 7); most of these deviations were participants receiving KP.2-adapted BNT162b2 too close to study vaccination (i.e., within 6 months) or not having previously received KP.2-adapted BNT162b2. Among participants in the historical control group, one participant in the 18–64 years of age group was excluded from the evaluable immunogenicity population because they did not have a valid and determinate immunogenicity result within 12 to 16 days after study vaccination.

**Figure 1:**
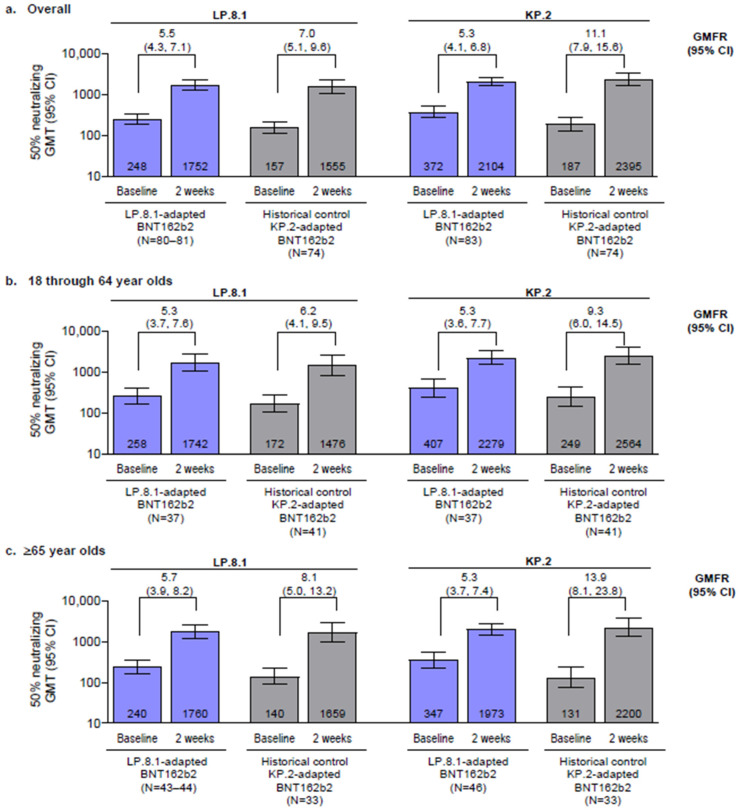
Serum-neutralizing GMTs and GMFRs (95% CIs) at baseline and 2 weeks after vaccination and from baseline to 2 weeks after vaccination, respectively, with LP.8.1-adapted BNT162b2 or KP.2-adapted BNT162b2 (historical controls) for LP.8.1 and KP.2: (**a**) overall, (**b**) in 18–64 -year-olds, and (**c**) ≥65-year-olds. Data are for the evaluable immunogenicity population (defined in Table S3). Values within the bars are GMTs. Values above the error bars are GMFRs (95% CI). GMFR, geometric mean fold rise; GMT, geometric mean titer; N, number of participants with valid and determinate GMT assay results.

Baseline GMTs (i.e., before vaccination) in the historical control group were notably lower against both LP.8.1 and KP.2 sublineages than those from the current study. At 2 weeks after vaccination, serum-neutralizing LP.8.1 and KP.2 GMTs increased from baseline in both the LP.8.1-adapted BNT162b2 vaccinated study group and historical control (participants who had received KP.2-adapted BNT162b2) overall and across both age groups (Figure 1). In the LP.8.1-adapted BNT162b2 vaccinated group in participants 18–64 and ≥65 years of age, GMTs (95% CI) at 2 weeks after vaccination were 1741.8 (1086.5, 2792.3) and 1760.2 (1186.3, 2611.6), respectively, against the LP.8.1 sublineage, and 2278.8 (1563.8, 3320.6) and 1973.1 (1443.4, 2697.2) against the KP.2 sublineage. In the historical comparator group that received the KP.2-adapted BNT162b2 vaccine, in participants 18–64 and ≥65 years of age, GMTs (95% CI) at 2 weeks after vaccination were 1475.7 (828.0, 2630.3) and 1658.8 (969.9, 2836.8), respectively, against the LP.8.1 sublineage, and 2563.9 (1582.1, 4154.9) and 2199.8 (1302.6, 3714.8) against the KP.2 sublineage.

In participants 18–64 and ≥65 years of age vaccinated with LP.8.1-adapted BNT162b2, GMFRs (95% CI) from before vaccination (baseline) to 2 weeks after vaccination were 5.3 (3.7, 7.6) and 5.7 (3.9, 8.2), respectively, against the LP.8.1 sublineage, and were 5.3 (3.6, 7.7) and 5.3 (3.7, 7.4) against KP.2. In the historical controls (in participants 18–64 and ≥65 years of age vaccinated with KP.2-adapted BNT162b2), GMFRs (95% CI) were 6.2 (4.1, 9.5) and 8.1 (5.0, 13.2), respectively, against the LP.8.1 sublineage and 9.3 (6.0, 14.5) and 13.9 (8.1, 23.8) against the KP.2 sublineage.

**Figure 2:**
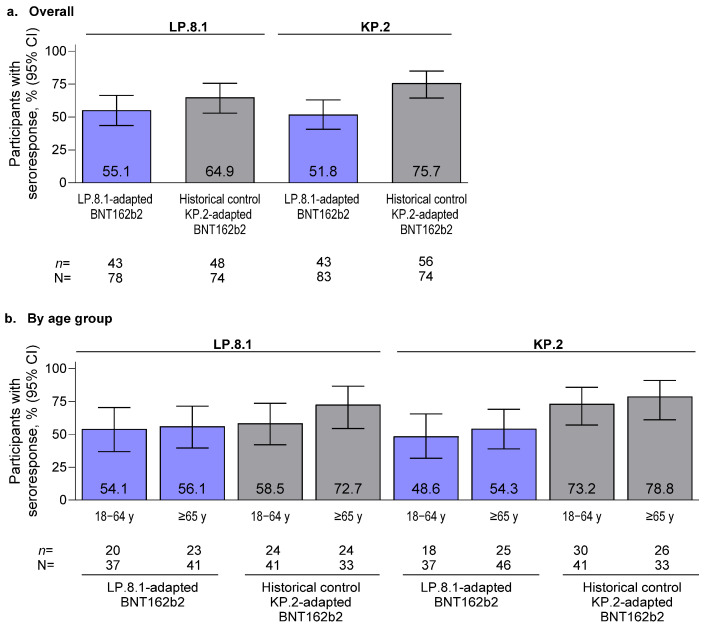
Percentage (95% CI) of participants achieving seroresponse against LP.8.1 and KP.2 at 2 weeks after vaccination with LP.8.1-adapted BNT162b2 or KP.2-adapted BNT162b2 (historical controls): (**a**) overall and (**b**) by age group. Data are for the evaluable immunogenicity population (defined in Table S3). N is the number of participants with valid and determinate assay results for the specified assay at both the pre-vaccination time point and at 2 weeks. These values are the denominators for percentage calculations. *n* is the number of participants with a seroresponse for the given assay at 2 weeks. Values above the error bars are the percentage of participants achieving seroresponse.

For both age groups and overall, the percentages of participants who achieved seroresponses were numerically higher in the historical controls (KP.2-adapted BNT162b2) compared with the LP.8.1-adapted BNT162b2 group against both LP.8.1 and KP.2 (Figure 2). Among those who received LP.8.1-adapted BNT162b2, 54.1% and 56.1% of participants 18–64 years of age and ≥65 years of age, respectively, achieved seroresponses against LP.8.1, whereas among those in the historical group (KP.2-adapted BNT162b2), 58.5% and 72.7% of participants 18–64 years of age and ≥65 years of age, respectively, achieved seroresponses. Among those who received LP.8.1-adapted BNT162b2, 48.6% and 54.3%, respectively, achieved seroresponses against KP.2, whereas 73.2% and 78.8% of participants 18–64 years of age and ≥65 years of age, respectively, in the historical group (KP.2-adapted BNT162b2) achieved seroresponses.

### 3.3. Safety

**Figure 3:**
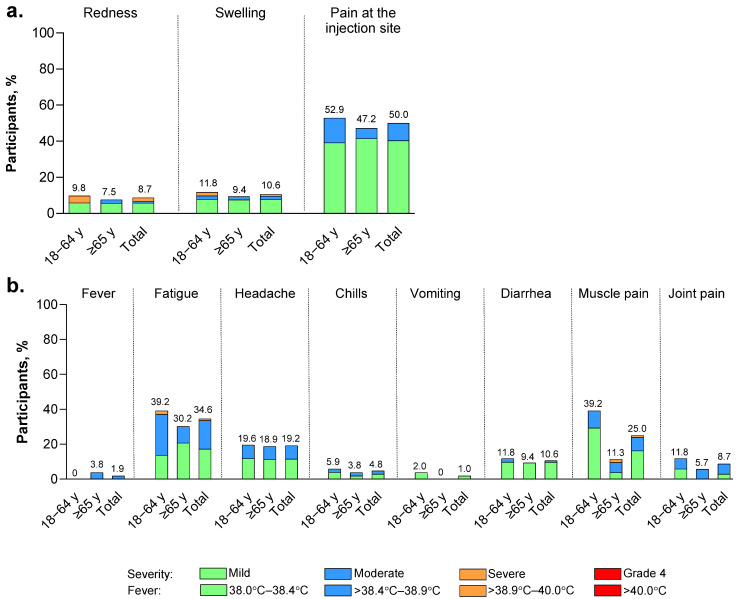
(**a**) Local reactions and (**b**) systemic events reported within 7 days of vaccination by age group, overall (safety population), and collected via electronic diary. Severity grading of the specific local reactions and systemic events is provided in Table S2. The values above the bars are the percentages of participants reporting an event of any severity. The numbers of participants in each age group were N = 51 (18–64 years of age) and N = 53 (≥65 years of age). There were no Grade 4 events or temperatures > 40.0 °C.

Local reactions reported within 7 days of receipt of LP.8.1-adapted BNT162b2 were generally mild to moderate in severity and occurred at similar frequencies in both age groups (Figure 3a). Pain at the injection site was the most common local reaction (52.9% of participants 18–64 years of age and 47.2% of participants ≥ 65 years of age). Three severe local reactions were reported: two (1.9%) cases of redness and one (1.0%) case of swelling, both in the 18–64 years of age group. The median durations of redness, swelling, and pain at the injection site were 3, 1, and 2 days, respectively.

Most systemic events occurring within 7 days of receipt of LP.8.1-adapted BNT162b2 were mild to moderate in severity (Figure 3b). Among participants 18–64 years of age, the most commonly reported systemic events were fatigue (39.2%), new or worsened muscle pain (39.2%), and headache (19.6%). Among participants ≥ 65 years of age, the most commonly reported systemic events were fatigue (30.2%), headache (18.9%), and new or worsened muscle pain (11.3%). Two severe systemic events were reported: one (1.0%) of fatigue in a participant 18–64 years of age and one (1.0%) of new or worsened muscle pain in a participant ≥ 65 years of age. No fevers *>* 38.4 °C were reported. Overall, the median duration of systemic events was 1 to 2 days.

As of the data cutoff, AEs within 2 weeks of receipt of LP.8.1-adapted BNT162b2 were reported in five (4.8%) participants, all of whom were 18–64 years of age; none were assessed by the investigator as related to the study vaccine (Table 2). No AEs were reported in participants ≥ 65 years of age. No SAEs, AESI, or cases of COVID-19 were reported in either age group.

## 4. Discussion

In this descriptive analysis from part of an ongoing phase 3 study, the monovalent Omicron LP.8.1-adapted BNT162b2 vaccine induced neutralizing responses against the LP.8.1 Omicron lineage at 2 weeks after vaccination in adults 18–64 years of age at high risk of severe COVID-19 and in older adults ≥ 65 years of age. LP.8.1-adapted BNT162b2 also induced neutralizing responses against another JN.1-descendant sublineage, KP.2, at 2 weeks after administration [28]. The observation of neutralizing responses against contemporaneous Omicron lineages is consistent with those from previous studies that evaluated other monovalent SARS-CoV-2 variant-adapted BNT162b2 vaccines against lineages that were prevalent when the studies were conducted [11,41,43].

Similar neutralizing antibody responses against both sublineages were observed 2 weeks after vaccination in both the LP.8.1-adapted BNT162b2 and historical control groups overall and across both age groups. In participants 18–64 years of age, GMFRs from baseline to 2 weeks after vaccination for LP.8.1-adapted BNT162b2 were similar to both LP.8.1 and KP.2 lineages (5.3 and 6.2, respectively), and slightly lower than the associated GMFRs for the KP.2-adapted BNT162b2 historical control group (6.2 and 9.3). In participants ≥ 65 years of age, GMFRs from baseline to 2 weeks after vaccination for LP.8.1-adapted BNT162b2 were generally similar to those observed in the younger group (5.7 and 5.3, respectively, against the LP.8.1 and KP.2 lineages), but lower than the associated GMFRs in participants ≥ 65 years of age in the KP.2-adapted BNT162b2 historical control group (8.1 and 13.9, respectively). These higher GMFRs in the KP.2-adapted BNT162b2 group reflect the lower baseline neutralizing titers against LP.8.1 and KP.2 lineages likely because of the longer median time since the last COVID-19 vaccination (13.3 vs. 9.5 months for participants 18–64 years of age and 21.4 vs. 9.3 months for participants ≥ 65 years of age in the KP.2-adapted BNT162b2 group vs. LP.8.1-adapted BNT162b2 groups, respectively) and lower likelihood of prior exposure to LP.8.1 and KP.2 strains. The higher percentages of participants achieving seroresponses against both LP.8.1 and KP.2 sublineages overall and by age group in the historical control group compared with the LP.8.1-adapted BNT162b2 group are again likely a consequence of the lower baseline neutralizing titers in the control group.

This descriptive analysis was not powered to detect differences in immune responses between the population vaccinated with study vaccine and the historical control; nevertheless, any observed differences (e.g., in GMTs, GMFRs and seroresponses) are not likely to be clinically significant. Importantly, the observed GMTs are similar or higher between the LP.8.1-adapted BNT162b2 recipients and the historical KP.2-adapted BNT162b2 group. Real-world evidence has shown that JN.1-adapted BNT162b2 and KP.2-adapted BNT162b2 both were effective against COVID-19, particularly against severe disease [41,44,45]. Notably, it is anticipated that forthcoming real-world evidence will support that the LP.8.1-adapted BNT162b2 vaccine will also provide protection against disease for contemporary homologous and closely related subvariants, such as the currently prevalent XFG subvariant, which is a recombinant derivative of Omicron LP.8.1 [46].

In this preliminary descriptive analysis of the LP.8.1-adapted BNT162b2 vaccine, few AEs and no AEs leading to withdrawal, SAEs, AESIs or deaths were reported within the 2-week assessment period for this analysis. Most local reactions and systemic events reported within 7 days of vaccination were mild to moderate in severity and consistent with the current US prescribing information for BNT162b2, as well as the reactogenicity profile previously reported for XBB.1.5-adapted, JN.1-adapted, and KP.2-adapted BNT162b2 vaccines [11,41,43,47].

Strengths of this study included the inclusion of participants regardless of SARS-CoV-2 seropositivity and inclusion of individuals with underlying medical conditions, reflecting real-world settings for those seeking vaccination. Limitations of this analysis include the short 2-week follow-up times for immunogenicity and safety, which also precluded completion of COVID-19 surveillance. Therefore, the durability of immune responses beyond 2 weeks cannot be assessed. However, previous neutralizing GMT results for KP.2-adapted and JN.1-adapted BNT162b2 show consistently robust responses at 14 days and at 1 month after vaccination [41]. Additionally, immunocompromised individuals were excluded, and although people living with HIV could participate, none were enrolled. Together with the small participant numbers, including for participants at risk of severe COVID-19, immune responses in these populations require further assessment. It is encouraging, however, that the observed immune responses in our study were similarly robust between younger adults who had at least one risk factor for severe COVID-19 and older adults. Similarly, longer-term safety was not assessed in this analysis, and there was a restricted number of study participants; AEs occurring beyond 2 weeks after vaccination or those occurring in a small percentage of vaccine recipients may therefore have been missed, including potential autoimmune reactions, neurologic or cardiac events, or menstrual disturbances. In the younger age group, approximately two-thirds of participants were of the female sex; it is not known if this imbalance affected immunogenicity or safety results. In addition, this study was not randomized but instead used a historical control group from a previous study that had differing inclusion criteria (i.e., had not previously received a COVID-19 vaccine or had received COVID-19 vaccination at least 150 days before without specification of the type of vaccine received) [41]. Lastly, this study evaluated US adults, and the findings may not be generalizable to other regions.

## 5. Conclusions

These descriptive 2-week findings describe the safety and immunogenicity of LP.8.1-adapted BNT162b2. The robust immune responses for LP.8.1-adapted BNT162b2 against contemporaneous strains are consistent with observations of immune responses from previous variant-adapted BNT162b2 vaccines and contribute to the dataset supporting the use of variant-adapted BNT162b2 vaccines. Vaccination remains important for protection against COVID-19, especially against severe disease, regardless of previous exposure to previous variants, either through vaccination or infection.

## Figures and Tables

**Table 1:** Baseline characteristics and demographics (safety population).

	LP.8.1-Adapted BNT162b2 Vaccine		
Characteristic	18–64 Years of Age (*N* = 51)	≥65 Years of Age (*N* = 53)	Total (*N* = 104)
Sex, *n* (%)			
Male	17 (33.3)	30 (56.6)	47 (45.2)
Female	34 (66.7)	23 (43.4)	57 (54.8)
Race, *n* (%)			
White	40 (78.4)	45 (84.9)	85 (81.7)
Black	5 (9.8)	3 (5.7)	8 (7.7)
Asian	4 (7.8)	3 (5.7)	7 (6.7)
Other/unknown/not reported	2 (3.9)	2 (3.8)	4 (3.8)
Ethnicity, *n* (%)			
Hispanic/Latino	18 (35.3)	14 (26.4)	32 (30.8)
Non-Hispanic/non-Latino	33 (64.7)	39 (73.6)	72 (69.2)
Age at vaccination, years			
Mean (SD)	49.2 (13.24)	72.3 (4.49)	61.0 (15.17)
Median (range)	54.0 (23, 64)	73.0 (65, 83)	65.0 (23, 83)
18–49 years, *n* (%)	19 (37.3)	0	19 (18.3)
50–64 years, *n* (%)	32 (62.7)	0	32 (30.8)
65–74 years, *n* (%)	0	35 (66.0)	35 (33.7)
≥75 years, *n* (%)	0	18 (34.0)	18 (17.3)
Baseline SARS-CoV-2 status, *n* (%)			
Positive ^a^	48 (94.1)	45 (84.9)	93 (89.4)
Medical history of COVID-19	22 (43.1)	18 (34.0)	40 (38.5)
Positive N-binding	46 (90.2)	42 (79.2)	88 (84.6)
Positive NAAT	1 (2.0)	2 (3.8)	3 (2.9)
Negative ^b^	3 (5.9)	8 (15.1)	11 (10.6)
Time from last dose of COVID-19 vaccine (received before the study) to the study vaccination, months ^c^			
Mean (SD)	15.1 (12.70)	12.3 (8.93)	13.7 (10.98)
Median (range)	10.0 (6.3, 55.9)	9.6 (6.5, 50.2)	9.7 (6.3, 55.9)
6 to <12 months	39 (76.5)	46 (86.8)	85 (81.7)
≥12 months	12 (23.5)	7 (13.2)	19 (18.3)
Body mass index ^d^, *n* (%)			
Underweight (<18.5 kg/m^2^)	1 (2.0)	1 (1.9)	2 (1.9)
Normal weight (18.5–24.9 kg/m^2^)	8 (15.7)	12 (22.6)	20 (19.2)
Overweight (25.0–29.9 kg/m^2^)	12 (23.5)	23 (43.4)	35 (33.7)
Obese (≥30.0 kg/m^2^)	30 (58.8)	17 (32.1)	47 (45.2)

N-binding, SARS-CoV-2 nucleoprotein-binding; NAAT, nucleic acid amplification test. ^a^ Positive N-binding antibody result at baseline, positive NAAT result at baseline, or medical history of COVID-19. ^b^ Negative N-binding antibody result at baseline, negative NAAT result at baseline, and no medical history of COVID-19. ^c^ Month was calculated as 28 days. ^d^ Body mass index was collected at enrollment to identify obese weight for higher-risk group stratification.

**Table 2:** Summary of unsolicited AEs through the 2-week follow-up period (safety population).

	LP.8.1-Adapted BNT162b2 Vaccine		
	18–64 Years of Age (*N* = 51) *n* (%)	≥65 Years of Age (*N* = 53) *n* (%)	Total (*N* = 104) *n* (%)
Any AE	5 (9.8)	0	5 (4.8)
Related ^a^	0	0	0
Severe	0	0	0
Life-threatening	0	0	0
Any SAE	0	0	0
Any nonserious AE	5 (9.8)	0	5 (4.8)
Related ^a^	0	0	0
Severe	0	0	0
Life-threatening	0	0	0
Any immediate AE ^b^	0	0	0
Any AE leading to withdrawal	0	0	0
Any AE of special interest ^c^	0	0	0
AE leading to death	0	0	0

AE, adverse event; SAE, serious adverse event. ^a^ Assessed by the investigator as related to the investigational vaccine. ^b^ Immediate AEs were AEs reported in the 30 min observation period after vaccination. ^c^ Protocol-specified AEs of special interest included confirmed diagnosis of myocarditis or pericarditis, and potential menstrual cycle disturbances.

## Data Availability

Upon request, and subject to review, Pfizer will provide the data that support the findings of this study. Subject to certain criteria, conditions, and exceptions, Pfizer may also provide access to the related individual de-identified participant data. See https://www.pfizer.com/science/clinical-trials/trial-data-and-results for more information.

## Supplementary Materials

The following supporting information can be downloaded at https://www.mdpi.com/article/10.3390/vaccines14040350/s1. Supplementary Text: Additional exclusion criteria, ethical study conduct, seroresponse, electrocardiogram, blood pressure, and pulse rate baseline assessments; Table S1: Risk factors for severe COVID-19 in participants 18–64 years of age [48]; Table S2: Severity grading for local reactions and systemic events; Table S3: Study populations; Table S4: Medical history by system organ class and preferred term (safety population); Table S5: Baseline characteristics and demographics for this study and historical control groups (evaluable immunogenicity population); and Figure S1: Study disposition for participants 18 through 64 and ≥65 years of age.

## Abbreviations

The following abbreviations are used in this manuscript:

AE	Adverse event
AESI	Adverse event of special interest
FDA	Food and Drug Administration
GMFR	Geometric mean fold rise
GMT	Geometric mean titer
LLOQ	Lower limit of quantitation
N-binding	SARS-CoV-2 nucleoprotein-binding
NAAT	Nucleic acid amplification test
SAE	Serious adverse event
VE	Vaccine effectiveness

## References

[1] Centers for Disease Control and Prevention COVID-19. Underlying Conditions and the Higher Risk for Severe COVID-19. https://www.cdc.gov/covid/hcp/clinical-care/underlying-conditions.html.

[2] Centers for Disease Control and Prevention (CDC) COVID 19. About COVID-19. https://www.cdc.gov/covid/about/index.html.

[3] World Health Organization Strategic and Operational Plan for Coronavirus Disease Threat Managment. https://cdn.who.int/media/docs/default-source/documents/epp/grt/draft_strategic-and-operational-plan-for-coronavirus-disease-threat-management.pdf?sfvrsn=30954d0d_4&download=true.

[4] Xiang Y., Zhang R., Qiu J., So H.C. (2025). Increased risk of hospitalization for various disorders after COVID-19 infection: A cohort study of the UK biobank spanning over a hundred disease categories. J. Microbiol. Immunol. Infect..

[5] Williamson E.J., Walker A.J., Bhaskaran K., Bacon S., Bates C., Morton C.E., Curtis H.J., Mehrkar A., Evans D., Inglesby P. (2020). Factors associated with COVID-19-related death using OpenSAFELY. Nature.

[6] Cummins L., Ebyarimpa I., Cheetham N., Tzortziou Brown V., Brennan K., Panovska-Griffiths J. (2021). Factors associated with COVID-19 related hospitalisation, critical care admission and mortality using linked primary and secondary care data. Influenza Other Respir. Viruses.

[7] Markov P.V., Ghafari M., Beer M., Lythgoe K., Simmonds P., Stilianakis N.I., Katzourakis A. (2023). The evolution of SARS-CoV-2. Nat. Rev. Microbiol..

[8] Le T.P., Abell I., Conway E., Campbell P.T., Hogan A.B., Lydeamore M.J., McVernon J., Mueller I., Walker C.R., Baker C.M. (2024). Modelling the impact of hybrid immunity on future COVID-19 epidemic waves. BMC Infect. Dis..

[9] Willett B.J., Grove J., MacLean O.A., Wilkie C., De Lorenzo G., Furnon W., Cantoni D., Scott S., Logan N., Ashraf S. (2022). SARS-CoV-2 Omicron is an immune escape variant with an altered cell entry pathway. Nat. Microbiol..

[10] Jacobs J.L., Haidar G., Mellors J.W. (2023). COVID-19: Challenges of viral variants. Annu. Rev. Med..

[11] Diya O., Gayed J., Lowry F.S., Ma H., Bangad V., Mensa F., Zou J., Xie X., Hu Y., Cutler M. (2025). A phase 2/3 trial to investigate the safety and immunogenicity of monovalent Omicron JN.1-adapted BNT162b2 COVID-19 vaccine in adults ≥18 years old. Vaccine.

[12] Mesle M.M.I., Brown J., Mook P., Katz M.A., Hagan J., Pastore R., Benka B., Redlberger-Fritz M., Bossuyt N., Stouten V. (2024). Estimated number of lives directly saved by COVID-19 vaccination programmes in the WHO European Region from December, 2020, to March, 2023: A retrospective surveillance study. Lancet Respir. Med..

[13] Semenzato L., Le Vu S., Botton J., Bertrand M., Jabagi M.-J., Drouin J., Cuenot F., Olié V., Dray-Spira R., Weill A. (2025). COVID-19 mRNA vaccination and 4-year all-cause mortality among adults aged 18 to 59 years in France. JAMA Network Open.

[14] Liu B., Stepien S., Dobbins T., Gidding H., Henry D., Korda R., Mills L., Pearson S.A., Pratt N., Vajdic C.M. (2023). Effectiveness of COVID-19 vaccination against COVID-19 specific and all-cause mortality in older Australians: A population based study. Lancet Reg. Health West. Pac..

[15] Ioannidis J.P.A., Pezzullo A.M., Cristiano A., Boccia S. (2025). Global estimates of lives and life-years saved by COVID-19 vaccination during 2020–2024. JAMA Health Forum.

[16] Anderer S. (2025). COVID-19 vaccines averted 2.5 million deaths, mostly among older adults. JAMA.

[17] US Food and Drug Administration COMIRNATY^®^ (COVID-19 Vaccine, mRNA) Package Insert. https://www.fda.gov/vaccines-blood-biologics/comirnaty.

[18] Sahin U., Muik A., Vogler I., Derhovanessian E., Kranz L.M., Vormehr M., Quandt J., Bidmon N., Ulges A., Baum A. (2021). BNT162b2 vaccine induces neutralizing antibodies and poly-specific T cells in humans. Nature.

[19] Bar-On Y.M., Goldberg Y., Mandel M., Bodenheimer O., Amir O., Freedman L., Alroy-Preis S., Ash N., Huppert A., Milo R. (2022). Protection by a fourth dose of BNT162b2 against Omicron in Israel. N. Engl. J. Med..

[20] Collie S., Nayager J., Bamford L., Bekker L.G., Zylstra M., Gray G. (2022). Effectiveness and durability of the BNT162b2 vaccine against Omicron sublineages in South Africa. N. Engl. J. Med..

[21] US Food and Drug Administration Coronavirus (COVID-19) Update: FDA Authorizes Moderna and Pfizer-BioNTech Bivalent COVID-19 Vaccines for Use as a Booster Dose in Younger Age Groups. https://web.archive.org/web/20221130214837/https://www.fda.gov/news-events/press-announcements/coronavirus-covid-19-update-fda-authorizes-moderna-and-pfizer-biontech-bivalent-covid-19-vaccines.

[22] European Medicines Agency Assessment Report: Comirnaty. https://www.ema.europa.eu/en/documents/variation-report/comirnaty-h-c-005735-ii-0140-epar-assessment-report-variation_en.pdf.

[23] US Food and Drug Administration COVID-19 Vaccines (2025–2026 Formula) for Use in the United States Beginning in Fall 2025. https://www.fda.gov/vaccines-blood-biologics/industry-biologics/covid-19-vaccines-2025-2026-formula-use-united-states-beginning-fall-2025.

[24] European Medicines Agency EMA Confirms Its Recommendation to Update the Antigenic Composition of Authorised COVID-19 Vaccines for 2024–2025. https://www.ema.europa.eu/en/documents/other/ema-confirms-its-recommendation-update-antigenic-composition-authorised-covid-19-vaccines-2024-2025_en.pdf.

[25] Link-Gelles R., Chickery S., Webber A., Ong T.C., Rowley E.A.K., DeSilva M.B., Dascomb K., Irving S.A., Klein N.P., Grannis S.J. (2025). Interim estimates of 2024–2025 COVID-19 vaccine effectiveness among adults aged ≥18 years—VISION and IVY Networks, September 2024-January 2025. MMWR Morb. Mortal. Wkly Rep..

[26] Pather S., Muik A., Rizzi R., Mensa F. (2023). Clinical development of variant-adapted BNT162b2 COVID-19 vaccines: The early Omicron era. Expert. Rev. Vaccines.

[27] US Food and Drug Administration FDA Approves and Authorizes Updated mRNA COVID-19 Vaccines to Better Protect Against Currently Circulating Variants. https://www.fda.gov/news-events/press-announcements/fda-approves-and-authorizes-updated-mrna-covid-19-vaccines-better-protect-against-currently.

[28] Chen L., Kaku Y., Okumura K., Uriu K., Zhu Y., Ito J., Sato K. (2025). Virological characteristics of the SARS-CoV-2 LP.8.1 variant. Lancet Infect. Dis..

[29] Abbad A., Lerman B., Ehrenhaus J., Monahan B., Singh G., Wilson A., Slamanig S., Aracena A., Lyttle N., Nardulli J. (2025). Antibody responses to SARS-CoV-2 variants LP.8.1, LF.7.1, NB.1.8.1, XFG and BA.3.2 following KP.2 monovalent mRNA vaccination. medRxiv.

[30] Jian F., Wang J., Yisimayi A., Song W., Xu Y., Chen X., Niu X., Yang S., Yu Y., Wang P. (2025). Evolving antibody response to SARS-CoV-2 antigenic shift from XBB to JN.1. Nature.

[31] GISAID Tracking of hCoV-19 Variants. https://gisaid.org/hcov19-variants/.

[32] Khoury D.S., Docken S.S., Subbarao K., Kent S.J., Davenport M.P., Cromer D. (2023). Predicting the efficacy of variant-modified COVID-19 vaccine boosters. Nat. Med..

[33] European Medicines Agency EMA Recommendation to Update the Antigenic Composition of Authorised COVID-19 Vaccines for 2025–2026. https://www.ema.europa.eu/en/documents/other/ema-recommendation-update-antigenic-composition-authorised-covid-19-vaccines-2025-2026_en.pdf.

[34] König S., Vaskyte U., Boesing M., Lüthi-Corridori G., Leuppi J.D. (2025). The role of comorbidities in COVID-19 severity. Viruses.

[35] Dryden-Peterson S., Kim A., Caniglia E.C., Joyce M.R., Rubins D., Kim A.Y., Fangman J., Baden L.R., Woolley A.E. (2025). Severe outcomes of COVID-19 among adults with increased risk conditions: A population-based observational study. PLoS ONE.

[36] Zhang J., Hou C., Chen W., Hu Y., Xu S., Liu H., Yang Y., Valdimarsdóttir U.A., Fang F., Song H. (2025). Comorbidity patterns associated with severe COVID-19 outcomes: A cohort study based on the UK Biobank. PLoS ONE.

[37] Abodunrin O.R., Olagunju M.T., Huang X., Wang J., Hu Z., Shen C. (2025). Regional risk factors associated with adverse outcomes of COVID-19 infection among the older adult: A systematic review and meta-analysis. J. Infect. Public Health.

[38] Centers for Disease Control and Prevention (CDC) Underlying Conditions and the Higher Risk for Severe COVID-19. https://www.cdc.gov/covid/hcp/clinical-care/underlying-conditions.html?CDC_AAref_Val=https://www.cdc.gov/coronavirus/2019-ncov/hcp/clinical-care/underlyingconditions.html.

[39] Winokur P., Gayed J., Fitz-Patrick D., Thomas S.J., Diya O., Lockhart S., Xu X., Zhang Y., Bangad V., Schwartz H.I. (2023). Bivalent Omicron BA.1-adapted BNT162b2 booster in adults older than 55 years. N. Engl. J. Med..

[40] Walsh E.E., Frenck R.W., Falsey A.R., Kitchin N., Absalon J., Gurtman A., Lockhart S., Neuzil K., Mulligan M.J., Bailey R. (2020). Safety and immunogenicity of two RNA-based Covid-19 vaccine candidates. N. Engl. J. Med..

[41] Diya O., Gayed J., Lowry F.S., Ma H., Bangad V., Mensa F., Zou J., Xie X., Hu Y., Cutler M. (2025). Safety and immunogenicity of monovalent Omicron KP.2-adapted BNT162b2 COVID-19 vaccine in adults: Single-arm substudy from a phase 2/3 trial. Infect. Dis. Ther..

[42] US Food and Drug Administration Guidance for Industry: Toxicity Grading Scale for Healthy Adult and Adolescent Volunteers Enrolled in Preventive Vaccine Clinical Trials. https://www.fda.gov/media/73679/download.

[43] Gayed J., Bangad V., Xu X., Mensa F., Cutler M., Türeci Ö., Şahin U., Modjarrad K., Swanson K.A., Anderson A.S. (2024). Immunogenicity of the monovalent Omicron XBB.1.5-adapted BNT162b2 COVID-19 vaccine against XBB.1.5, BA.2.86, and JN.1 sublineages: A phase 2/3 trial. Vaccines.

[44] Appaneal H.J., Lopes V.V., Puzniak L., Zasowski E.J., Jodar L., McLaughlin J.M., Caffrey A.R. (2024). Early effectiveness of the BNT162b2 KP.2 vaccine against COVID-19 in the US Veterans Affairs Healthcare System. medRxiv.

[45] Hansen C.H., Lassauniere R., Rasmussen M., Moustsen-Helms I.R., Valentiner-Branth P. (2025). Effectiveness of the BNT162b2 and mRNA-1273 JN.1-adapted vaccines against COVID-19-associated hospitalisation and death: A Danish, nationwide, register-based, cohort study. Lancet Infect. Dis..

[46] World Health Organization (2025). WHO TAG-VE Risk Evaluation for SARS-CoV-2 Variant Under Monitoring: XFG.

[47] (2025). COMIRNATY (COVID-19 vaccine mRNA). Full Prescribing Information.

[48] Prasad V., Makary M.A. (2025). An Evidence-Based Approach to Covid-19 Vaccination. N. Engl. J. Med..

