# Supplementary material for: A Study to Investigate the Safety and Immunogenicity of Monovalent Omicron LP.8.1-Adapted BNT162b2 COVID-19 Vaccine in Adults ≥ 65 Years of Age and High-Risk Adults 18–64 Years of Age (Preliminary Results)
Source: Vaccines (Basel). 2026 Apr 15;14(4):350. doi: 10.3390/vaccines14040350 (PMC13120441; doi:10.3390/vaccines14040350)
Supplement: Supplementary file 1 [file vaccines-14-00350-s001.zip › vaccines-4138550-Figure S1.pdf]

Figure S1. Study disposition for participants 18–64 and ≥65 years of age.

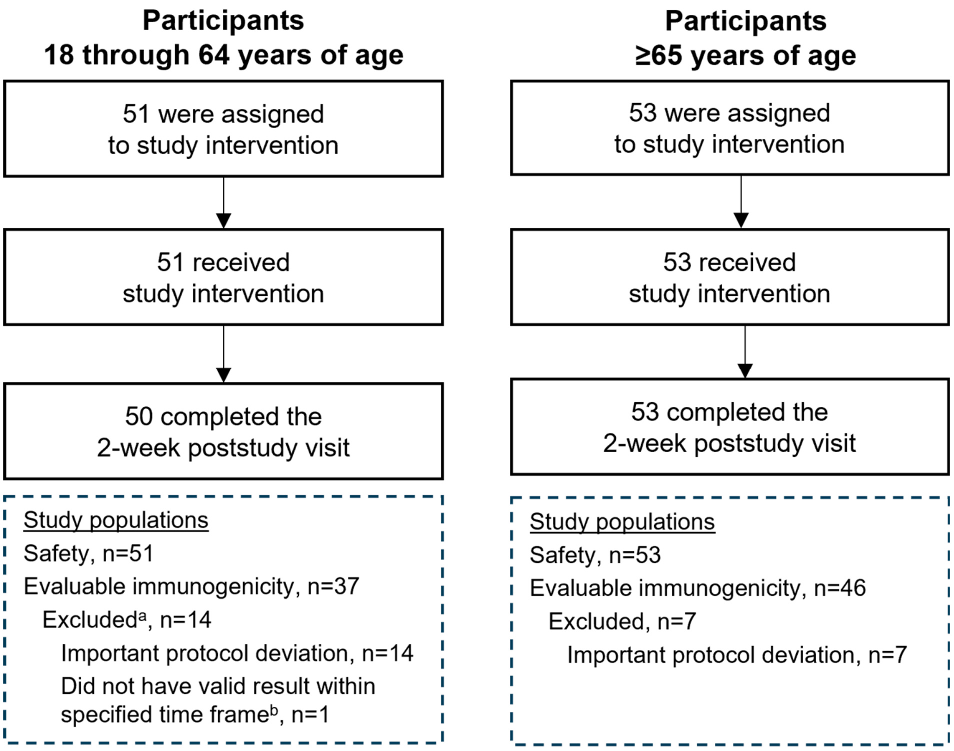

[a]Participants could have more than 1 reason for exclusion from a population. [b]Did not have ≥1 valid and determinate immunogenicity result within 12–16 days after study vaccination.
