# Supplementary material for: A Study to Investigate the Safety and Immunogenicity of Monovalent Omicron LP.8.1-Adapted BNT162b2 COVID-19 Vaccine in Adults ≥ 65 Years of Age and High-Risk Adults 18–64 Years of Age (Preliminary Results)
Source: Vaccines (Basel). 2026 Apr 15;14(4):350. doi: 10.3390/vaccines14040350 (PMC13120441; doi:10.3390/vaccines14040350)
Supplement: Supplementary file 1 [file vaccines-14-00350-s001.zip › vaccines-4138550-Supplementary Text.pdf]

## SUPPLEMENTARY TEXT

### Additional Exclusion Criteria

Participants were also excluded if they had received treatment with known systemic immunosuppressant medications or radiotherapy within 60 days of enrollment or planned receipt through study conclusion, received blood or plasma products for the treatment or prevention of COVID-19 from 60 days before administration of LP.8.1-adapted BNT162b2 or planned to receive these throughout the study, or received at least a 14-day course of systemic corticosteroids (≥20 mg/day of prednisone or its equivalent) within 28 days before enrollment or planned to receive these through study conclusion.

### Ethical Study Conduct

The study was conducted in accordance with consensus ethical principles derived from international guidelines including the Declaration of Helsinki Council, the Council for International Organizations of Medical Sciences International Ethical Guidelines, applicable International Council for Harmonization of Technical Requirements for Pharmaceutical for Human Use Good Clinical Practice Guidelines, and other applicable laws and regulations. The protocol and informed consent documents were approved by an institutional review board/ethics committee. All participants provided written informed consent before enrollment.

### Seroresponse

Seroresponse was defined as achieving a ≥4-fold rise in titers from baseline; if the baseline value was below the lower limit of quantitation (LLOQ) the post-vaccination measure of ≥4 × LLOQ was considered seroresponse.

### Electrocardiogram, Blood Pressure, and Pulse Rate Baseline Assessments

Participants 18–64 years of age underwent baseline electrocardiogram (ECG), blood pressure, and pulse rate assessments at baseline. Standard 12-lead ECGs were collected at the first study visit using an ECG system that automatically calculates the heart rate (HR) and measures PR interval, QT interval, QT corrected by heart rate by Fridericia’s formula (QTcF), and QRS complex. Participants with a concerning or clinically significant ECG abnormality were not to receive study vaccination. Any clinically significant changes from the baseline 1 ECG were considered potential AEs and were to be evaluated further, as clinically warranted.
