# Supplementary material for: A Study to Investigate the Safety and Immunogenicity of Monovalent Omicron LP.8.1-Adapted BNT162b2 COVID-19 Vaccine in Adults ≥ 65 Years of Age and High-Risk Adults 18–64 Years of Age (Preliminary Results)
Source: Vaccines (Basel). 2026 Apr 15;14(4):350. doi: 10.3390/vaccines14040350 (PMC13120441; doi:10.3390/vaccines14040350)
Supplement: Supplementary file 1 [file vaccines-14-00350-s001.zip › vaccines-4138550-Table S1.pdf]

Table S1. Risk factors for severe COVID-19 in participants 18–64 years of age[a]

- Asthma
- Type I diabetes mellitus
- Type II diabetes mellitus
- Heart conditions (eg, previous coronary artery disease or heart failure but excluding cardiomyopathy, myocarditis and pericarditis)
- HIV infection
- Mental health conditions limited to mood disorders, including depression
- Parkinson disease
- Obesity (body mass index ≥30 kg/m^2)
- Smoking, current and former

[a]Risk factors were developed utilizing conditions listed by the Centers for Disease Control and Prevention.[1]

[1] Prasad V, Makary MA. An Evidence-Based Approach to Covid-19 Vaccination. N Engl J Med. 2025;392(24):2484-2486.
