# Supplementary material for: A Study to Investigate the Safety and Immunogenicity of Monovalent Omicron LP.8.1-Adapted BNT162b2 COVID-19 Vaccine in Adults ≥ 65 Years of Age and High-Risk Adults 18–64 Years of Age (Preliminary Results)
Source: Vaccines (Basel). 2026 Apr 15;14(4):350. doi: 10.3390/vaccines14040350 (PMC13120441; doi:10.3390/vaccines14040350)
Supplement: Supplementary file 1 [file vaccines-14-00350-s001.zip › vaccines-4138550-Table S2.pdf]

Table S2. Severity grading for local reactions and systemic events

	Mild	Moderate	Severe	Potentially life threatening (Grade 4)
Local reactions				
Pain at the injection site	Does not interfere with activity	Interferes with activity	Prevents daily activity	Emergency department visit or hospitalization for severe pain
Redness	>2.0 cm to 5.0 cm (5 to 10 measuring device units)	>5.0 cm to 10.0 cm (11 to 20 measuring device units)	>10 cm (≥21 measuring device units)	Necrosis or exfoliative dermatitis
Swelling	>2.0 cm to 5.0 cm (5 to 10 measuring device units)	>5.0 cm to 10.0 cm (11 to 20 measuring device units)	>10 cm (≥21 measuring device units)	Necrosis
Systemic events				
Diarrhea	2–3 loose stools in 24 hours	4–5 loose stools in 24 hours	≥6 loose stools in 24 hours	Emergency department visit or hospitalization for severe diarrhea
Vomiting	1–2 times in 24 hours	≥2 times in 24 hours	Requires intravenous hydration	Emergency department visit or hospitalization for hypotensive shock
Headache	Does not interfere with activity	Some interference with activity	Prevents daily routine activity	Emergency department visit or hospitalization for severe headache
Fatigue	Does not interfere with activity	Some interference with activity	Prevents daily routine activity	Emergency department visit or hospitalization for severe fatigue
Chills	Does not interfere with activity	Some interference with activity	Prevents daily routine activity	Emergency department visit or hospitalization for severe chills
Muscle pain (new or worsened)	Does not interfere with activity	Some interference with activity	Prevents daily routine activity	Emergency department visit or hospitalization for severe new or worsened muscle pain
Joint pain (new or worsened)	Does not interfere with activity	Some interference with activity	Prevents daily routine activity	Emergency department visit or hospitalization for severe new or worsened joint pain
Fever	38.0°C to 38.4°C	>38.4°C to 38.9°C	>38.9°C to 40.0°C	>40.0°C
