# Supplementary material for: A Study to Investigate the Safety and Immunogenicity of Monovalent Omicron LP.8.1-Adapted BNT162b2 COVID-19 Vaccine in Adults ≥ 65 Years of Age and High-Risk Adults 18–64 Years of Age (Preliminary Results)
Source: Vaccines (Basel). 2026 Apr 15;14(4):350. doi: 10.3390/vaccines14040350 (PMC13120441; doi:10.3390/vaccines14040350)
Supplement: Supplementary file 1 [file vaccines-14-00350-s001.zip › vaccines-4138550-Table S3.pdf]

Table S3. Study populations

Participant Analysis Set	Description
Assigned	All participants who are assigned a randomization number in the IRT system.
Safety	All participants who received study intervention.
Evaluable immunogenicity	All eligible assigned participants who received the study intervention to which they were assigned, had at least 1 valid and determinate immunogenicity result from the blood sample collected within an appropriate window after vaccination, and had no other important protocol deviation as determined by the clinician.

IRT, interactive response technology.
