# Supplementary material for: A Study to Investigate the Safety and Immunogenicity of Monovalent Omicron LP.8.1-Adapted BNT162b2 COVID-19 Vaccine in Adults ≥ 65 Years of Age and High-Risk Adults 18–64 Years of Age (Preliminary Results)
Source: Vaccines (Basel). 2026 Apr 15;14(4):350. doi: 10.3390/vaccines14040350 (PMC13120441; doi:10.3390/vaccines14040350)
Supplement: Supplementary file 1 [file vaccines-14-00350-s001.zip › vaccines-4138550-Table S4.pdf]

Table S4. Medical history by system organ class and preferred term[a] (safety population)

	LP.8.1-adapted BNT162b2 vaccine	
	18–64 years of age (N=51)	≥65 years of age (N=53)
**System organ class**	***n* (%)**	***n* (%)**
Any medical history	50 (98.0)	53 (100.0)
Blood and lymphatic system disorders	2 (3.9)	1 (1.9)
Cardiac disorders	4 (7.8)	8 (15.1)
Atrial fibrillation	0	3 (5.7)
Coronary artery disease	2 (3.9)	4 (7.5)
Myocardial infarction	1 (2.0)	2 (3.8)
Ventricular extrasystoles	1 (2.0)	2 (3.8)
Congenital, familial, and genetic disorders	1 (2.0)	3 (5.7)
Ear and labyrinth disorders	3 (5.9)	3 (5.7)
Vertigo	2 (3.9)	1 (1.9)
Endocrine disorders	7 (13.7)	9 (17.0)
Hypothyroidism	5 (9.8)	8 (15.1)
Eye disorders	5 (9.8)	16 (30.2)
Cataract	0	5 (9.4)
Glaucoma	0	5 (9.4)
Myopia	2 (3.9)	1 (1.9)
Presbyopia	2 (3.9)	4 (7.5)
Gastrointestinal disorders	12 (23.5)	20 (37.7)
Constipation	2 (3.9)	4 (7.5)
Gastroesophageal reflux disease	9 (17.6)	9 (17.0)
Hiatus hernia	2 (3.9)	2 (3.8)
Inguinal hernia	0	5 (9.4)
General disorders and administration site conditions	4 (7.8)	6 (11.3)
Drug intolerance	3 (5.9)	3 (5.7)
Fatigue	1 (2.0)	2 (3.8)
Hepatobiliary disorders	7 (13.7)	5 (9.4)
Cholelithiasis	6 (11.8)	2 (3.8)
Immune system disorders	19 (37.3)	15 (28.3)
Drug hypersensitivity	13 (25.5)	8 (15.1)
Seasonal allergy	9 (17.6)	11 (20.8)
Infections and infestations	28 (54.9)	20 (37.7)
Appendicitis	4 (7.8)	2 (3.8)
COVID-19	22 (43.1)	16 (30.2)
Tonsillitis	1 (2.0)	3 (5.7)
Injury, poisoning, and procedural complications	3 (5.9)	7 (13.2)
Investigations	7 (13.7)	4 (7.5)
Cardiac murmur	2 (3.9)	1 (1.9)
SARS-CoV-2 test positive	1 (2.0)	3 (5.7)
Metabolism and nutrition disorders	38 (74.5)	47 (88.7)
Dyslipidemia	8 (15.7)	10 (18.9)
Glucose tolerance impaired	2 (3.9)	4 (7.5)
Hypercholesterolemia	5 (9.8)	10 (18.9)
Hyperlipidemia	4 (7.8)	13 (24.5)
Obesity	24 (47.1)	11 (20.8)
Overweight	5 (9.8)	14 (26.4)
Type 2 diabetes mellitus	13 (25.5)	10 (18.9)
Vitamin D deficiency	5 (9.8)	2 (3.8)
Musculoskeletal and connective tissue disorders	16 (31.4)	21 (39.6)
Arthralgia	1 (2.0)	3 (5.7)
Arthritis	2 (3.9)	1 (1.9)

	LP.8.1-adapted BNT162b2 vaccine	
**System organ class**	**18–64 years of age (N=51)** ***n* (%)**	**≥65 years of age (N=53)** ***n* (%)**
Back pain	6 (11.8)	3 (5.7)
Intervertebral disc degeneration	1 (2.0)	2 (3.8)
Osteoarthritis	5 (9.8)	10 (18.9)
Osteoporosis	1 (2.0)	4 (7.5)
Osteopenia	0	3 (5.7)
Spinal osteoarthritis	2 (3.9)	1 (1.9)
Spondylitis	0	3 (5.7)
Neoplasms benign, malignant, and unspecified[b]	7 (13.7)	15 (28.3)
Hemangioma of skin	1 (2.0)	2 (3.8)
Seborrheic keratosis	1 (2.0)	2 (3.8)
Uterine leiomyoma	3 (5.9)	4 (7.5)
Nervous system disorders	10 (19.6)	15 (28.3)
Diabetic neuropathy	1 (2.0)	3 (5.7)
Headache	2 (3.9)	1 (1.9)
Migraine	3 (5.9)	4 (7.5)
Neuropathy peripheral	3 (5.9)	1 (1.9)
Pregnancy, puerperium, and perinatal conditions	0	1 (1.9)
Psychiatric disorders	25 (49.0)	18 (34.0)
Anxiety	14 (27.5)	8 (15.1)
Attention deficit hyperactivity disorder	10 (19.6)	0
Depression	16 (31.4)	9 (17.0)
Generalised anxiety disorder	3 (5.9)	0
Insomnia	7 (13.7)	8 (15.1)
Major depression	3 (5.9)	1 (1.9)
Renal and urinary disorders	7 (13.7)	7 (13.2)
Nephrolithiasis	2 (3.9)	2 (3.8)
Reproductive system and breast disorders	10 (19.6)	16 (30.2)
Artificial menopause	2 (3.9)	2 (3.8)
Benign prostatic hyperplasia	1 (2.0)	9 (17.0)
Erectile dysfunction	1 (2.0)	4 (7.5)
Respiratory, thoracic, and mediastinal disorders	23 (45.1)	10 (18.9)
Asthma	13 (25.5)	1 (1.9)
Chronic obstructive pulmonary disease	3 (5.9)	0
Obstructive sleep apnea syndrome	3 (5.9)	5 (9.4)
Rhinitis allergic	4 (7.8)	1 (1.9)
Sleep apnea syndrome	1 (2.0)	2 (3.8)
Skin and subcutaneous tissue disorders	12 (23.5)	8 (15.1)
Actinic keratosis	2 (3.9)	3 (5.7)
Social circumstances	13 (25.5)	20 (37.7)
Ex-tobacco user	1 (2.0)	4 (7.5)
Postmenopause	12 (23.5)	13 (24.5)
Tobacco user	1 (2.0)	3 (5.7)
Surgical and medical procedures	24 (47.1)	32 (60.4)
Appendicectomy	4 (7.8)	2 (3.8)
Caesarean section	6 (11.8)	2 (3.8)
Cataract operation	0	3 (5.7)
Cholecystectomy	5 (9.8)	2 (3.8)
Female sterilization	5 (9.8)	1 (1.9)
Fracture treatment	0	3 (5.7)
Hysterectomy	4 (7.8)	5 (9.4)
Inguinal hernia repair	0	4 (7.5)
Intervertebral disc operation	0	3 (5.7)
Knee arthroplasty	2 (3.9)	4 (7.5)

	LP.8.1-adapted BNT162b2 vaccine	
	18–64 years of age (N=51)	≥65 years of age (N=53)
**System organ class**	***n* (%)**	***n* (%)**
Mammoplasty	0	3 (5.7)
Salpingectomy	2 (3.9)	2 (3.8)
Tonsillectomy	1 (2.0)	4 (7.5)
Vascular disorders	21 (41.2)	35 (66.0)
Essential hypertension	9 (17.6)	14 (26.4)
Hypertension	11 (21.6)	19 (35.8)
Peripheral vascular disorder	1 (2.0)	2 (3.8)

[a]Occurring in ≥2 participants overall. [b]Including cysts and polyps.
