# Supplementary material for: A Study to Investigate the Safety and Immunogenicity of Monovalent Omicron LP.8.1-Adapted BNT162b2 COVID-19 Vaccine in Adults ≥ 65 Years of Age and High-Risk Adults 18–64 Years of Age (Preliminary Results)
Source: Vaccines (Basel). 2026 Apr 15;14(4):350. doi: 10.3390/vaccines14040350 (PMC13120441; doi:10.3390/vaccines14040350)
Supplement: Supplementary file 1 [file vaccines-14-00350-s001.zip › vaccines-4138550-Table S5.pdf]

Table S5. Baseline characteristics and demographics for the study and historical control groups (evaluable immunogenicity population)

Characteristic	LP.8.1-adapted BNT162b2			Historical control (KP.2-adapted BNT162b2)		
	18–64 years of age (N=37)	≥65 years of age (N=46)	Total (N=83)	18–64 years of age (N=41)	≥65 years of age (N=33)	Total (N=74)
Sex, *n* (%)						
Male	13 (35.1)	26 (56.5)	39 (47.0)	19 (46.3)	16 (48.5)	35 (47.3)
Female	24 (64.9)	20 (43.5)	44 (53.0)	22 (53.7)	17 (51.5)	39 (52.7)
Race, *n* (%)						
White	28 (75.7)	38 (82.6)	66 (79.5)	31 (75.6)	23 (69.7)	54 (73.0)
Black	4 (10.8)	3 (6.5)	7 (8.4)	6 (14.6)	5 (15.2)	11 (14.9)
Asian	3 (8.1)	3 (6.5)	6 (7.2)	3 (7.3)	4 (12.1)	7 (9.5)
Other/Unknown/Not Reported	2 (5.4)	2 (4.3)	4 (4.8)	1 (2.4)	1 (3.0)	2 (2.7)
Ethnicity, *n* (%)						
Hispanic/Latino	17 (45.9)	14 (30.4)	31 (37.3)	1 (2.4)	5 (15.2)	6 (8.1)
Non-Hispanic/non-Latino	20 (54.1)	32 (69.6)	52 (62.7)	39 (95.1)	28 (84.8)	67 (90.5)
Age at vaccination, years						
Mean (SD)	49.2 (13.6)	72.4 (4.5)	62.1 (15.06)	47.0 (10.07)	70.9 (4.49)	57.7 (14.40)
Median (range)	55.0 (23, 64)	72.5 (65, 83)	67.0 (23, 83)	45.0 (24, 63)	70.0 (65, 83)	61.0 (24, 83)
Baseline SARS-CoV-2 status, *n* (%)						
Positive[a]	35 (94.6)	39 (84.8)	74 (89.2)	38 (92.7)	30 (90.9)	68 (91.9)
Medical history of COVID-19	20 (54.1)	18 (39.1)	38 (45.8)	18 (43.9)	11 (33.3)	29 (39.2)
Positive N-binding	33 (89.2)	36 (78.3)	69 (83.1)	38 (92.7)	30 (90.9)	68 (91.9)
Positive NAAT	1 (2.7)	2 (4.3)	3 (3.6)	2 (4.9)	4 (12.1)	6 (8.1)
Negative[b]	2 (5.4)	7 (15.2)	9 (10.8)	3 (7.3)	3 (9.1)	6 (8.1)
Time from last dose of COVID-19 vaccine (received prior to the study) to the study vaccination, months[c]						
n	37	46	83	38	30	68
Mean (SD)	9.1 (1.43)	9.2 (1.26)	9.2 (1.33)	18.5 (11.33)	22.3 (12.27)	20.2 (11.82)
Median (range)	9.5 (6.3, 11.1)	9.3 (6.5, 11.1)	9.4 (6.3, 11.1)	13.3 (5.5, 45.5)	21.4 (6.0, 47.3)	13.8 (5.5, 47.3)
<6 months	0	0	0	1 (2.4)	1 (3.0)	2 (2.7)
≥6 to <12 Months	37 (100.0)	46 (100.0)	83 (100.0)	13 (31.7)	6 (18.2)	19 (25.7)

>12 Months	0	0	0	24 (58.5)	23 (69.7)	47 (63.5)
Body mass index[d], *n* (%)						
Underweight (<18.5 kg/m^2)	1 (2.7)	1 (2.2)	2 (2.4)	1 (2.4)	0	1 (1.4)
Normal weight (≥18.5–24.9 kg/m^2)	6 (16.2)	9 (19.6)	15 (18.1)	4 (9.8)	10 (30.3)	14 (18.9)
Overweight (≥25.0–29.9 kg/m^2)	9 (24.3)	21 (45.7)	30 (36.1)	9 (22.0)	9 (27.3)	18 (24.3)
Obese (≥30.0 kg/m^2)	21 (56.8)	15 (32.6)	36 (43.4)	27 (65.9)	14 (42.4)	41 (55.4)

N-binding, SARS-CoV-2 nucleoprotein-binding; NAAT, nucleic acid amplification test.

[a]Positive N-binding antibody result at baseline, positive NAAT result at baseline, or medical history of COVID-19.

[b]Negative N-binding antibody result at baseline, negative NAAT result at baseline, and no medical history of COVID-19.

[c]Month was calculated as 28 days.

[d]Body mass index was collected to identify obese weight for higher risk group stratification rather than medical history.
